# Effect of integrated English hybrid learning for undergraduate nursing students on students’ self-perceived communication competence and communication apprehension: A quasi-experiment study

**DOI:** 10.1016/j.heliyon.2024.e35350

**Published:** 2024-07-26

**Authors:** Hartiah Haroen, Fanny Adistie, Hana Rizmadewi Agustina, Neti Juniarti

**Affiliations:** a Department of Community Health Nursing, Faculty of Nursing, Universitas Padjadjaran, 45363, Sumedang, Indonesia; b Department of Fundamental and Pediatric Nursing, Faculty of Nursing, Universitas Padjadjaran, 45363, Sumedang, Indonesia

**Keywords:** Communication apprehension, Communication competence, English for nursing purposes, Hybrid learning, Nursing students

## Abstract

**Aim:**

To demonstrate the self-perceived communication competence and communication apprehension of nursing students before and after taking English for Nursing courses.

**Background:**

Despite the growing demand for English as a foreign language courses for nursing students in Indonesia, relatively little research has been conducted to investigate whether these courses meet students’ specific learning needs to increase communication competency.

**Design:**

Quasi-experiment.

**Methods:**

A total of 173 undergraduate nursing students participated and had access to the mixed-methods learning system of integrated English hybrid learning from August through December 2021. The learning methods involved lectures, tutorials, and project-based learning on multiple platforms, such as LiVE Unpad, WhatsApp, and Zoom. Communication competence and apprehension in the English Language were assessed using the Self-Perceived Communication Competence and Communication Apprehension questionnaires via Google Form, and were analyzed using the paired *t*-test and ANOVA.

**Results:**

The findings suggest that English for Nursing courses significantly increased self-perceived communication competence and communication apprehension among nursing students (*p* < 0.001 for both). A variance test revealed favorability for self-perceived communication apprehension in groups that enjoyed learning English and for communication apprehension in groups that read English texts frequently and rarely.

**Conclusions:**

Integrated English hybrid learning for undergraduate nursing students effectively increased their self-perceived communication competence and communication apprehension in undergraduate nursing students. However, this approach cannot be applied to all nursing education levels. A combination of the current and new approaches, as suggested by educators and policymakers, should make it applicable to the desired level of nursing education.

## Highlights

• Students in the study demonstrated an increase in SPCC and CA after the course.
• This study suggests that multiple methods in an EFN course improves students' communication comprehension and skills.
• The study also explores baseline group differences in SPCC and CA, showing relations to reading English texts and enjoyment of learning English.
• A multimethod course, including lectures, tutorials, and project-based learning, enhances nursing students' English language skills.

## 1 Introduction

Globally, the need for nurses is relatively high. The World Health Organization estimates that by 2030, nine million nurses and midwives are needed globally to achieve Sustainable Development Goal 3 on health and well-being [1]. However, global healthcare executives and policymakers are concerned about the severity of the nurse workforce crisis and its influence on health in the United States and other developed countries [2]. English proficiency is a mandatory requirement for nurses and other healthcare professionals to become an international health workforce [3]. As such, integrating English into nursing education is necessary to prepare English language competency for nursing graduates to prepare for international placement [2].

Good English competency is essential for nursing students as English is an international language. Competency includes integrating basic English skills (speaking, listening, reading, and writing) within the scope of nursing and health in general to prepare students to provide nursing care to English-speaking patients as well as to communicate with nursing and other health care professionals [4]. Students also need English to support learning activities, as most nursing textbooks and other references are available in English. Furthermore, nursing care based on research evidence or evidence-based practice (EBP) requires students to critically review published research literature, most of which is in English [5]. Nonetheless, there are some challenges in learning English for non-English-speaking nursing students, such as in Indonesia. A study in Indonesia concluded that challenges experienced by students in learning English include the application of grammar, structure, and tense in nursing fields and the lack of role models of lecturers in English speaking and communication [6]. A similar study in Taiwan found that nursing-related English vocabulary, pronunciation, and accent were challenges experienced by nurses [4].

It has been argued that achieving good competency in English does not merely depend on students. Nursing lecturers play a significant role in promoting student engagement by developing instructional strategies tailored to their current needs and learning objectives [3]. The opportunities for students to improve their English communication both in the classroom and in the clinical setting are essential for lectures and faculty [7]. Some strategies facilitate nursing students in achieving learning outcomes in English subjects, such as modifying the curriculum and learning strategies and encouraging nursing students to use English in their daily activities [6]. Thus, teaching and learning innovations and strategies should be delivered by utilizing available resources and facilities. Using multiple methods, teaching and learning can support students’ learning outcomes in English courses.

Students’ competence in communicating in English is influenced by their motivation and abilities. Self-perceived communication competence (SPCC) is a determinant factor in the development of English communication competence [8]. Moreover, communication apprehension (CA) has been recognized as one of the most significant factors in reducing communication willingness [9]. Despite evidence suggesting that high SPCC scores correlate with better persistence, less anxiety, deeper material comprehension, and higher grades in English [10], gaps remain in the understanding of how multi-method learning approaches affect these variables. This study, therefore, aimed to measure changes in SPCC and CA among nursing students before and after they participated in a multi-method English for Nursing course, addressing these gaps, and evaluating the effectiveness of such educational interventions.

## 2 Methods

### 2.1 Study design

The study design was a quasi-experiment with pre-and post-interventions to evaluate the effectiveness of integrated English hybrid learning for undergraduate nursing student courses. This study followed the Transparent Reporting of Evaluation Non-randomized Design (TREND) guidelines. This study was approved by the Research Ethics Committee of Padjadjaran, Indonesia (793/UN6. KEP/EC/2021). The students were provided with information regarding the study in a written format using Google Forms. The information included the aim of the study, the voluntarism of participation, and the privacy and confidentiality of the involvement in the study. All collected data were kept confidential through anonymization techniques, with the removal of personal identifiers that could identify the subjects. Pseudonymization was also applied by assigning each subject a unique code, ensuring that the data remained relevant for analysis without compromising privacy. The students were informed that there would not be any consequences of any kind if they refused to participate in the study or withdrew from their participation at any time.

### 2.2 Sampling and participation

This study was conducted in the Undergraduate Program, Faculty of Nursing, Universitas Padjadjaran. The population is undergraduate students and A total of 180 undergraduate students enrolled in an English for Nursing (EFN) course were invited to participate in this study. The sample size for this study was determined through a power analysis, considering the primary variable of interest, namely self-perceived communication competence and communication apprehension, and based on an anticipated effect size of −0.483, as derived from previous relevant studies [8]. Power analysis was carried out with a significance level (α) of 0.05, and a desired power (1-β) of 0.80. Using these parameters, the calculated sample size required for this study was 68 participants. It is important to note that this estimation considers potential variability in the population and allows for sufficient statistical power to detect meaningful effects. To ensure the robustness of the findings and compensate for any attrition during the study, we recruited all students. Only 173 students (96.1 %) provided consent to participate in the study. The remaining 3.9 % of participants did not provide consent and did not participate in the study.

### 2.3 Procedure

The study was conducted between August and December 2021. The students were asked to complete a Google form to measure their SPCC and CA before the course began. Haroen et al. [11] implemented an approach similar to the multi-method course in Indonesia by Haroen et al. [11]. The learning methods included lectures (flipped classroom methods and communicative language teaching [CLT]), tutorials, and projects (see Table 1). The main learning platform used in this study was LiVE Unpad, a Learning Management System (LMS) provided by Universitas Padjadjaran combined with WhatsApp and Zoom applications. Asynchronous activities were carried out through the LiVE Unpad, and synchronous activities used zoom meetings. After the students completed the EFN course, SPCC and CA were measured using Google Forms.

**Table 1:** The multi-methods of English for Nursing (EFN) course.

Method	Description
Lectures	Lectures were delivered synchronously and asynchronously using the flipped classroom method. Zoom meeting was used to deliver lectures synchronously. In contrast, LiVE Unpad delivered the teaching videos and learning materials asynchronously from academic lecturers. Student engagement is maintained using the chat feature and forum in LiVE Unpad and WhatsApp groups. The CLT method is also applied to joint learning activities with a native speaker from Texas, The USA.
Tutorial	Students are divided into small groups, and each group had one tutor. The tutorial activity aims to improve listening, reading, writing, and speaking skills. In addition, the CLT method is also applied to this activity by emphasizing the learning process of using language for communication rather than the process of imitating, drilling, memorizing, and studying language structures. The CLT method makes students learn foreign languages well through grammar and translation and continue practicing to form habits [12]. Students also learn languages through interacting and communicating.
Project-based	Each group of students made a video that performed a nursing assessment of the patient (specific anamneses, e.g., demographics data, chief complaints, health history, and activity daily living). The group also were assigned to develop a role-play on the nursing procedure.

### 2.4 Measurements

The measurements were adapted from the SPCC instruments developed by Lockley and Subekti and the CA developed by Subekti [8,13]. The instruments utilized in this study included 15 items: Students' Perceptions of Communication Competence (SPCC) and 15 items Communication Apprehension (CA) [8]. SPCC measures students' perceptions of how effectively they can communicate, encompassing various dimensions such as verbal and nonverbal communication skills, confidence in expressing ideas, and interpersonal effectiveness. The SPCC includes subscales that specifically assess written communication and presentation skills. On the other hand, CA measures students' anxiety levels related to communicating in front of others. Subscales within the CA instrument delve into distinct contexts of communication apprehension, including public speaking, group discussions, and interpersonal interactions [8]. Participants responded to the questionnaire using four possible responses. For items on SPCC, the responses were: “Strongly agree” (4), “Agree” (3), “Disagree” (2), and “Strongly disagree” (1) [8]. In contrast, for items on CA, “Strongly agree” (1), “Agree” (2), “Disagree” (3), and “Strongly disagree” (4). The instruments were translated forward-backward into Bahasa Indonesia and English by a certified linguist. Validity and reliability tests were conducted with 54 students from different years of study. Construct validity was reinforced by the finding that the identified factors reflected the expected dimensions of the variable (r > 0.60). Moreover, the reliability showed excellent internal consistency (McDonald's omega = 0.94) [14].

### 2.5 Data analysis

Jamovie version 2.2.5 was used to analyze the data. Data analysis was conducted using both the univariate and bivariate methods. Univariate analysis was performed to describe the characteristics of the participants and bivariate analysis was used to assess the outcomes. The Shapiro-Wilk test was conducted to determine data normality. The difference between the scores’ pre- and post-EFN course means were analyzed using a paired *t*-test. In addition, ANOVA was conducted to evaluate the variance among the groups of studies.

## 3 Results

### 3.1 Characteristics of participant

A total of 173 students participated in this study. Most of the students were female and were aged 19 years and over, with a mean of 19.0 years (SD 0.522). Most students have Grade Point Averages (GPA) of more than 3.50 with a mean of 3.51 (SD 0.269). More than half of the students (52 %) attended private English lessons. The majority of participants sometimes read English texts and enjoyed learning English (76,45 %). The participants’ characteristics are presented in Table 2.

**Table 2:** Characteristic of participants (N = 173).

Characteristic	Frequency (n)	Percentage (%)
**Age (years)**	Mean = 19.0	SD = 0.522
**< 19**	21	12.1
**≥ 19**	152	87.9
**Gender**		
**Male**	11	6.4
**Female**	162	93.6
**GPA (/4.00)**	Mean = 3.51	SD = 0.269
**< 3.25**	27	15.6
**3.25**–**3.50**	56	32.4
> **3.50**	90	52.0
**Private English course**		
**Yes**	90	52.0
**No**	77	44.5
**Ongoing**	6	3.5
**Read English text**		
**Often**	25	20.3
**Sometimes**	94	76.4
**Never**	4	3.3
**Enjoy English learning**		
**Yes**	167	96.5
**No**	6	3.5

A descriptive test was conducted. SD = standard deviation; GPA = Grade Point Average.

### 3.2 Effect of integrated English hybrid learning on SPCC and CA among nursing students

**Fig. 1:**
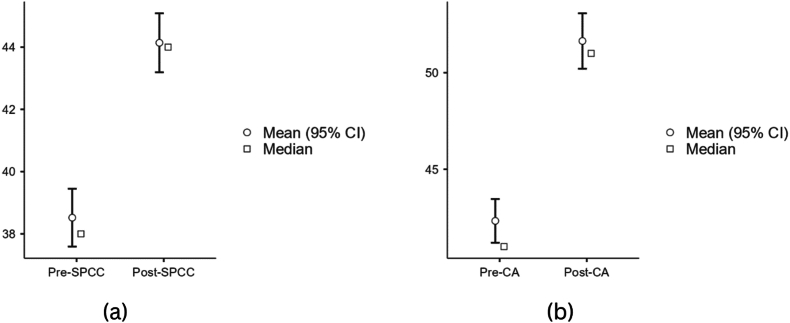
Descriptive plot comparison of SPCC and CA between pre-and post-EFN course.

Before the integrated English hybrid learning for undergraduate nursing student courses, students had an SPCC with a mean of 38.5 (SD 6.36) and CA 42.3 (SD 7.60). After the integrated English hybrid learning, the SPCC and CA increased to 44.1 (SD 6.36) and 51.6 (SD 9.62) after integrated English hybrid learning. Therefore, integrated English hybrid learning significantly increased the SPCC and CA among nursing students (*p* < 00.001 for both). Table 3 describes the study outcomes statistically, and Fig. 1a and b shows plot of the differences between the mean SPCC and CA pre- and post-EFN courses, respectively.

**Table 3:** Comparison of SPCC and CA between Pre- and Post-EFN course.

Variable	Pre Mean (SD)	Post Mean (SD)	MD	95 % CI (Lower; Upper)	t	*p-*value
SPCC	38.5 (6.23)	44.1 (6.36)	−5.62	−6.44; −4.80	−13.5	<0.001
CA	42.3 (7.60)	51.6 (9.62)	−9.31	−11.2; −7.38	−9.50	<0.001

A paired *t*-test was conducted. Normality test using Shapiro Wilk for SPCC and CA (*p =* 0.366, *p* = 0.933, respectively); SD = standard deviation; MD = mean difference.

### 3.3 Variance test between groups to SPCC and CA

Based on the variance test in the demographic and baseline groups, there were differences in CA between the groups who often, rarely, and never read English texts (*p* = 0.031).The groups who often and rarely were had higher CA score than the other, with a mean 52.7 (SD 9.98). Moreover, there was also a difference in SPCC between the group that enjoyed and did not enjoy learning English (*p* = 0.001).The group that enjoyed learning English was had higher CA score than the other group, with a mean 44.4 (SD 6.14). Meanwhile, the other variables did not show any differences between the groups. The details of the variations between the groups are shown in Table 4.

**Table 4:** Variance analysis of characteristics of participants toward SPCC and CA after the INSERT course.

Variable		Outcome	N	Mean (SD)	F	df1	df2	*p*
Gender	Male	SPCC	11	43.2 (4.45)	−0.514	N/A	N/A	0.608^a^
	Female		162	44.2 (6.48)				
	Male	CA	11	52.6 (10.29)	3.53	N/A	N/A	0.724^a^
	Female		162	51.6 (9.61)				
Age (years)	<19	SPCC	21	45.8 (7.37)	1.29	N/A	N/A	0.200^a^
	≥19		152	43.9 (6.20)				
	<19	CA	21	48.8 (9.76)	−1.44	N/A	N/A	0.151^a^
	≥19		152	52.0 (9.57)				
GPA (/4.00)	<3.25	SPCC	27	43.4 (6.24)	1.749	2	70.7	0.181^b^
	3.25–3.50		56	43.1 (6.05)				
	>3.50		90	45.0 (6.53)				
	<3.25	CA	27	52.9 (10.31)	0.262	2	68.2	0.770^b^
	3.25–3.50		56	51.6 (9.36)				
	>3.50		90	51.3 (9.65)				
Private English course	Yes	SPCC	90	44.3 (6.06)	0.0486	2	13.6	0.953^b^
	No		77	44.0 (6.72)				
	Ongoing		6	43.5 (7.29)				
	Yes	CA	90	50.6 (9.73)	1.0713	2	13.3	0.370^b^
	No		77	52.7 (9.11)				
	Ongoing		6	53.5 (14.01)				
Read English text	Often	SPCC	25	44.6 (6.08)	0.544	2	7.93	0.601^b^
	Sometimes		94	43.4 (6.46)				
	Never		4	41.5 (6.14)				
	Often	CA	25	51.1 (10.58)	5.427	2	8.42	0.031^b^
	Sometimes		94	52.7 (9.98)				
	Never		4	40.8 (6.70)				
Enjoy English learning	Yes	SPCC	167	44.4 (6.14)	3.278	N/A	N/A	0.001^a^
	No		6	36.0 (7.72)				
	Yes	CA	167	51.6 (9.41)	−0.653	N/A	N/A	0.515^a^
	No		6	54.2 (15.51)				

¶ independent *t*-test statistic; SD = standard deviation; GPA = Grade point average; N/A = not available.

a ^)^an independent *t*-test was conducted.

b One-way ANOVA was conducted.

## 4 Discussion

### 4.1 Principal finding

Despite being the largest group of health professionals in the health industry, the world continues to experience a shortage of nurses. The International Council of Nurses identified that nurses' shortages were further provoked by the COVID-19 pandemic, which exacerbated the problem, with 89 % of these shortages concentrated in low- and lower-middle-income nations [15]. It was also emphasized that nations in the WHO regions of Africa, Southeast Asia, and the Eastern Mediterranean all had significant shortage gaps [15]. With this issue in hand, the urgency to have globally competent nurses is even more prominent. To meet this demand, tertiary education systems should prepare nursing students to compete globally through the development of an international program [16]. As English is the most recognized and accepted global language, proficiency in English has become necessary for nursing students [17]. However, the nursing curriculum often fails to accommodate an appropriate syllabus to develop students' English skills. As such, this study aimed to evaluate nursing students’ SPCC and CA in English as a second language before and after receiving integrated English hybrid learning.

Many studies have analyzed the need for an English as a foreign language course for nursing students. However, the present study is believed to be the first pre-and post-intervention study of an English-for-nursing course that uses integrated English hybrid learning to improve students' SPCC and CA in Indonesia. The current study found that both SPCC and CA increased in nursing students after they received integrated English hybrid learning. This study's findings contradict a previous study that showed SPCC is negatively associated with CA. According to the model developed by Chen et al. [18], regarding the willingness to communicate among students in mobile medical consultations, SPCC may not be affected solely by CA. However, SPCC is also influenced by perceived social presence (PSP). Therefore, PSP may have a greater influence on SPCC among students in this study.

Further analysis showed the favorability of SPCC in the group that enjoyed learning English and CA in the groups that often and sometimes read English texts. These findings are consistent with a study that found that students who understood English learning materials were more likely to have a more positive perception of fluency [8]. In contrast, distressful experiences while learning English produce anxiety while practicing the language [8]. The value of English courses in this context needs to be considered in light of these findings. While the increase in CA might initially be likely to detract from the benefits of the course, the simultaneous increase in SPCC indicates that students feel more confident and anxious simultaneously. This suggests that, although the course for some level effectively enhances competence, it should be designed to respond to and mitigate student anxiety. Future iterations of the course could incorporate elements specifically aimed at reducing CA, such as creating a relaxed environment, supportive class system, and interaction, as well as providing positive non-judgmental reinforcement.

The present study suggested that students were more inclined to have better assurance of their language skills after experiencing various methods of learning. Although multiple methods were not used, several previous studies have found that learning methods that actively engage students in learning English improve students' ability and confidence in speaking English. Munfadlila who conducted a study in Indonesia, concluded that the speaking English skills of the students improved after they were involved in a series of roleplays [19]. Another quasi-experimental study in the Indonesian context found that roleplays and simulations positively improved students’ speaking skills [20].

There are several methods for nurses to learn English. If students learn English from a variety of sources, such as dictionaries, movies, songs, and articles from the Internet, they are likely to be more proficient in basic language skills such as writing, listening, reading, and speaking [21]. English courses were also found to be more helpful when students could learn from reading materials, group projects, presentations, and discussion forums in the nursing field [22]. Although it has been agreed upon by previous studies that students dominantly perceive simulations as improving their speaking skills, other communicative abilities are important to develop professional competencies in nursing [[18], [19], [20], [21], [22]]. The present study used a mixed-method learning strategy in which students participated in roleplay, lectures, and tutorials. This follows the research conducted by Habil, Othman, and Kahar (2016), who found that most nurses need English competency to write instructions, speak with different roles in the hospital, receive and understand face-to-face instructions, and read and follow safety standards and measures [23]. When implementing different exercises to improve communicative skills, nurses can gain beneficial outcomes such as better information flow and higher patient and family satisfaction [24].

### 4.2 The implication to practice

To ensure that the pressing global demand for high-quality nurses is met, nursing students are expected to develop their English language skills. Nurses are responsible for providing care to patients regardless of their culture, religion, linguistic ability, or ethnic background [25]. Further emphasis was placed when medical and health tourism became a global phenomenon, where patients sought treatment outside of their home country, where the required care may be more attainable [26]. Therefore, having English communication skills is a valuable asset for nursing students because it is pivotal to build high-quality relationships with patients from different backgrounds [27].

English is often taught as a foreign language that enables students to communicate globally, use professional resources and materials written in English, and provide better nursing care to all patients. English has become a universal language used in professional communication by healthcare workers in both overseas and domestic settings. Therefore, it is crucial for nursing students to learn English skills that are useful in the workplace [28]. English for Nursing courses also provides opportunities for students to promote their personal development, since they may enhance their academic abilities and professional competencies [28]. In terms of personality and nursing competence, Indonesian nurses are able to compete with nurses from other nations. On the contrary, a lack of English proficiency may prevent them from expanding their international careers [26]. Educational background, language, and cultural competencies are major requirements when preparing internationally qualified nurses because they can influence their professional practice [28,29]. Therefore, training in English communication skills through English for Nursing courses during undergraduate studies and professional practice is a mandatory agenda.

Although the present study found that the English for Nursing course increased both SPCC and CA, students in groups with more preferences and exposure to the English language did better in communication. English for Nursing is a course taught for specific purposes to inform students of nursing competency in English. Typically, the objective of the course is to provide comprehensive information to nursing students to challenge their ability to practice English, to provide specific materials based on nursing students' needs, and to increase students’ motivation and courage to express their ideas and opinions on nursing-related topics in English [6]. Thus, how nursing educational institutions can improve the English communication skills of their students, whether speaking, writing, listening, or reading, constitutes the primary challenge in implementing the course.

Studies on English for Nursing courses have focused primarily on the situation of international nursing students in countries where English is the native language. The discourse on learning English as a foreign language for nursing students using a mixed-methods approach is still in its infancy. This study contributes to the emergence of new conversations regarding effective learning models that can be adopted, particularly by nursing education institutions intended to internationalize their campuses.

### 4.3 Limitations

This study utilizes a quasi-experimental design to assess the impact of an English communication program on nursing students, which is a novel contribution in the context of nursing education. This study demonstrates a significant improvement in students' communication competence, ⁠⁠provides actionable insights into how nursing education programs can integrate communication training, offering practical recommendations for educators and institutions. However, some limitations to this study should be acknowledged. First, it did not further analyze why students' CA also appeared to have increased. In his study, Subekti stated that, as the learning process progresses, students’ SPCC is expected to improve [8]. When the SPCC improves, the reported CA is expected to reduce. Second, the absence of a control group in our experimental design limits our ability to draw definitive causal inferences between SPCC and CA. This design may contribute to the contradictory findings reported, as we cannot definitively rule out external factors influencing the observed changes. Third, this study was conducted during the COVID-19 global pandemic, which created a limited interactive learning experience that is essential to exercising communicative skills. The results gathered in the present study may have differed if the multi-method approach for the learning process of EFN allowed for higher interactivity. Finally, this study was not able to provide any solid qualitative feedback from students regarding integrated English hybrid learning. On the other hand, this study affirmed that a multi-method course for students learning English as a foreign language can be an alternative when singular approaches are insufficient.

## 5 Conclusions

It can be argued that integrating several methods of learning English among nursing students improved their communication comprehension and skills. The effectiveness of lectures, tutorials, and project-based learning facilitates students’ exploration and enhancement of their English language skills. These findings are vital because nursing students form the future of the global nursing workforce. Proficiency and fluency in English are essential assets for nursing students to work abroad and join the global nursing workforce. Nevertheless, since integrated English hybrid learning is only focused on undergraduate nursing students, it may not be effective for postgraduate education because of the different characteristics and needs of the students. For this research to progress, more studies are needed to determine the best way to teach English communication skills at all stages of nursing schools.

## Ethical statement

All participants gave their informed consent before they participated in the study. The study was conducted in accordance with the Declaration of Helsinki, and protocol of this study was approved by the Research Ethics Committee of Universitas Padjadjaran, Indonesia (793/UN6. KEP/EC/2021).

## Funding sources

This research was funded by Hibah Inovasi Pembelajaran Daring (HIPDU) with funding number 2042/UN6. RKT/Kep/HK/2021, Universitas Padjadjaran. APC was funded by the 10.13039/501100014823Directorate of Research and Community Engagement of 10.13039/501100015690Universitas Padjadjaran.

## Data availability

To protect the privacy and confidentiality of our participants, we were required to restrict the data and limit access to authorized individuals only. The data supporting the findings of this study are available from the corresponding author (Hartiah Haroen), upon reasonable request.

## CRediT authorship contribution statement

**Hartiah Haroen:** Writing – review & editing, Writing – original draft, Visualization, Validation, Supervision, Software, Resources, Project administration, Methodology, Investigation, Funding acquisition, Formal analysis, Data curation, Conceptualization. **Fanny Adistie:** Writing – review & editing, Writing – original draft, Visualization, Validation, Supervision, Software, Resources, Methodology, Investigation, Funding acquisition, Formal analysis, Data curation. **Hana Rizmadewi Agustina:** Writing – review & editing, Validation. **Neti Juniarti:** Writing – review & editing, Validation.

## Declaration of competing interest

The authors declare the following financial interests/personal relationships which may be considered as potential competing interests: Hartiah Haroen reports financial support was provided by 10.13039/501100015690Padjadjaran University.
