# Supplementary material for: Effect of integrated English hybrid learning for undergraduate nursing students on students’ self-perceived communication competence and communication apprehension: A quasi-experiment study
Source: Heliyon. 2024 Jul 26;10(15):e35350. doi: 10.1016/j.heliyon.2024.e35350 (PMC11334622; doi:10.1016/j.heliyon.2024.e35350)
Supplement: Multimedia component 1 [file mmc1.docx]

# QUESTIONNAIRE

# ENGLISH VERSION OF THE QUESTIONNAIRE:

**Appendix 1: Participants’ response for questionnaire regarding SPCC**

| **Strongly Agree** | **Agree** | **Disagree** | **Strongly Disagree** |
| --- | --- | --- | --- |

1. I am able to give directions to my favorite restaurant in English to a person sitting next to me.

|  |  |  |  |
| --- | --- | --- | --- |

1. I am able to translate verbal expressions in Indonesian to English within my group.

|  |  |  |  |
| --- | --- | --- | --- |

1. I am able to play roles in English in front of the class (eg. ordering food in a restaurant).

|  |  |  |  |
| --- | --- | --- | --- |

1. I am able to give a short introduction of myself without bringing any notes.

|  |  |  |  |
| --- | --- | --- | --- |

1. I feel that I am able to speak in English without too much effort.

|  |  |  |  |
| --- | --- | --- | --- |

1. I am certain I can attempt to make myself understandable when speaking English.

|  |  |  |  |
| --- | --- | --- | --- |

1. I know I am able to speak English with familiar words even when the words are inaccurate.

|  |  |  |  |
| --- | --- | --- | --- |

1. I am able to speak without nervousness.

|  |  |  |  |
| --- | --- | --- | --- |

1. I feel alright when speaking English in front of a class this size.

|  |  |  |  |
| --- | --- | --- | --- |

1. I am able to speak English in public.

|  |  |  |  |
| --- | --- | --- | --- |

1. I feel more comfortable speaking English in public.

|  |  |  |  |
| --- | --- | --- | --- |

1. I am able to speak English more fluently than before.

|  |  |  |  |
| --- | --- | --- | --- |

1. I feel that my English is good enough.

|  |  |  |  |
| --- | --- | --- | --- |

1. I know I can give a speech in English if I prepared myself.

|  |  |  |  |
| --- | --- | --- | --- |

1. I can try to give a speech if I have a draft ready.

|  |  |  |  |
| --- | --- | --- | --- |

**Appendix 2: Participants’ response for CA questionnaire**

1. I feel tense during presentations.

|  |  |  |  |
| --- | --- | --- | --- |

1. Even now, I am still traumatic of my failure in doing presentations.

|  |  |  |  |
| --- | --- | --- | --- |

1. I am worried that I am using English strangely.

|  |  |  |  |
| --- | --- | --- | --- |

1. I know my English is bad so it is difficult for me to speak English and I lose my confidence.

|  |  |  |  |
| --- | --- | --- | --- |

1. I feel embarrassed to speak in public.

|  |  |  |  |
| --- | --- | --- | --- |

1. I feel a little nervous if I speak English incorrectly in public.

|  |  |  |  |
| --- | --- | --- | --- |

1. I still feel nervous even though I have given several speeches.

|  |  |  |  |
| --- | --- | --- | --- |

1. I never feel confident with myself when I speak English in class.

|  |  |  |  |
| --- | --- | --- | --- |

1. Not understanding what the teacher said in English is a scary thing for me.

|  |  |  |  |
| --- | --- | --- | --- |

1. I start to get panicked when I have to speak in English class with no preparation.

|  |  |  |  |
| --- | --- | --- | --- |

1. I feel self-conscious when I speak English in front of other students.

|  |  |  |  |
| --- | --- | --- | --- |

1. I feel nervous and confused when I speak English in my English class.

|  |  |  |  |
| --- | --- | --- | --- |

1. I feel nervous when I don’t understand everything my English teacher said.

|  |  |  |  |
| --- | --- | --- | --- |

1. I feel weary of the number of rules that are needed to be learnt when speaking English.

|  |  |  |  |
| --- | --- | --- | --- |

1. I tremble when I know that my teacher will point at me in English class.

|  |  |  |  |
| --- | --- | --- | --- |

**APPENDIX A**

**Questionnaire**

For each question, circle the number that best describes yourself (1 = strongly disagree to 5 = strongly agree).

1. What is your name?
2. My high school English class had us meet native English speakers.

1 2 3 4 5

1. I am able to read in English well.

1 2 3 4 5

1. I think about grammar before I speak.

1 2 3 4 5

1. I am certain I know the usage of who, which, that, what, whatever, whoever, whichever.

1 2 3 4 5

1. I am good at listening in English.

1 2 3 4 5

1. I am certain I know how to express comparison in English.

1 2 3 4 5

8a) I am confident in my ability to read in English,

1 2 3 4 5

8b) Please explain your answer.

9a) I feel confident in my ability to write in English.

1 2 3 4 5

9b) Please explain your answer.

10a) I am confident in my ability to listen in English.

1 2 3 4 5

10b) Please explain your answer.

11a) I feel confident in my ability to speak English.

1 2 3 4 5

11b) Please explain your answer.

1. I am certain I know how to make sentences using present tense.

1 2 3 4 5

1. I am certain I know how to use conditionals.

1 2 3 4 5

1. I am able to listen well in English.

1 2 3 4 5

1. I am certain I know how to use future perfect continuous tense. (I’ll have finished reading this book by the end of this month)

1 2 3 4 5

1. I am able to speak English well.

1 2 3 4 5

1. I am able to write in English.

1 2 3 4 5

1. I am certain I know how to write passive sentences in English.

1 2 3 4 5

1. I am good at speaking English.

1 2 3 4 5

1. I am certain I know how to use past perfect tense.

1 2 3 4 5

1. I feel nervous when I speak English with the ELI teacher.

1 2 3 4 5

1. I feel nervous when speaking with native speakers aside from the ELI teacher, such as exchange students or when I am on vacation.

1 2 3 4 5

1. I am good at reading in English.

1 2 3 4 5

1. I can write in English well.

1 2 3 4 5

1. I am certain I know how to write sentences using past tense (“He walked home”).

1 2 3 4 5

1. I am certain I know how to use modals (may, must, could, should, would, have to, be able to).

1 2 3 4 5

**Appendix B**

**KEPT verbal assessment rubric**

|  | **Pronounciation**  Think about:   - **Word degrees** - **Sentence level: ability to combine or connect sounds within or between words.** - **Emphasis, rhythm, and intonation** - **Accent** | **Fluency**  Think about:   - **Automation: ability to formulate sentences quickly and ease of speaking** - **Speaking speed** - **Hesitation and pauses** | **Grammar/Lexicon**  Think about:   - **Proper language forms** - **Suitability of vocabulary** - **Displays ability to use (or attempt to use) different grammar structure and vocabulary according to context.** - **Proper word choice and collocation** | **Conversational skills**  Think about:   - **Participation and ease of interaction (alternating, responding, asking questions, introducing new steps, paraphrasing, *hedging*^[[1]](#footnote-1)^)** |
| --- | --- | --- | --- | --- |
|  | **Unacceptable pronounciation**   - Very thick accent that hinders communication - Only using phonology and rhythm such as in katakana; words don’t combine | **Unacceptable fluency**   - Stammering while speaking - Stopping, often illegible - Communication close to impossible | **Unacceptable grammar and lexicon** | **Unacceptable conversation skills**   - No awareness of other speakers; can speak but not like conversing - Impossible to communicate |
| **0**  **~ 0.5** |  |  | - No evidence of grammatical knowledge - Knows several words and uses them separately - Unable to share simple ideas - Communication is impossible |  |
| **1.0**  **~ 1.5** | **Low pronounciation**   - Uses Katakana-like pronounciation; doesn’t combine words - May experience difficulty in understanding others. | **Low fluency**   - Tense, slow, unreasonable. - Unreasonable stammering. - Unreasonably long pauses. - Difficult to communicate. | **Low grammar and lexicons**   - Evidence of limited grammatical knowledge - Limited vocabulary, unskilled use - Little to no attempt at complex vocabulary or grammar. | **Low conversation skills**   - Does not start interaction. - Largely monologous - May share turns speaking but does not combine ideas well, or gives many explanations. |
|  |  |  | - Able to convey ideas, but may be difficult to understand. |  |
| **2.0**  **~ 2.5** | **Medium pronounciation**   - Have not mastered challenging English sounds, but is largely understandable - Tries to combine words but may still make mistakes on emphasis | **Medium fluency**   - Hesitant - Unreasonable stammering and lulls in conversation may exist without hindering communication - May use excessive fillers | **Medium grammar and lexicon**   - Relies heavily on small amounts of grammar and vocabulary to express ideas - Shows little to no evidence of controlling challenging grammar or vocabulary | **Medium communication skills**   - Awareness of taking turns - Maintains interaction by responding without unreasonable pauses or stops - Shows agreement or disagreement of others’ opinions |
| **3.0**  **~ 3.5** | **Good pronounciation**   - May not have mastered all English sounds, but has good control in sentence emphasis and intonation. - Accent does not hinder understanding, consistently combines words. | **Good fluency**   - Occasional misuse of fillers, may still stammer but does not hinder understanding. | **Good grammar and lexicon**   - Shows evidence of controlling complex grammar or vocabulary and attempts to use many forms. - May make many mistakes, but is still understandable. | **Good communication skills**   - Confident - Responds to others properly - Ability to direct conversations - Shows ability to negotiate meanings quickly and naturally - May start paraphrasing and clarifying in a lower stage. |
| **4**  **~**  **?** | **Very good pronounciation**   - Mastered many English sounds - Accent does not hinder communication | **Very good fluency**   - Conversation goes smoothly with few obstacles - Uses fillers and lexical pauses effectively - Some stammering may occur, but looks natural and smooth. | **Very good grammar and lexicons**   - Shows very good use of grammar and vocabulary - Mistakes may happen, but does not hinder meaning - May use idioms and collocations properly | **Very good communication skills**   - Very confident and natural - May ask others to broaden view - Negotiates, hold and release speaking turns properly - Explains how their own idea relates to others’ idea, interacts with ease. |

# INDONESIAN-TRANSLATED VERSION OF THE QUESTIONNAIRE:

**Lampiran 1: Jawaban Peserta untuk butir kuesioner terkait SPCC mereka**

| **Sangat Setuju** | **Setuju** | **Tidak Setuju** | **Sangat Tidak Setuju** |
| --- | --- | --- | --- |

1. Saya dapat memberikan petunjuk arah ke restoran favorit saya dalam Bahasa Inggris ke teman yang duduk di sebelah saya.

|  |  |  |  |
| --- | --- | --- | --- |

1. Saya dapat menerjemahkan ungkapan verbal dalam Bahasa Indonesia ke Bahasa Inggris di kelompok saya.

|  |  |  |  |
| --- | --- | --- | --- |

1. Saya dapat bermain peran dalam Bahasa Inggris di depan kelas (misalnya memesan makanan di restoran).

|  |  |  |  |
| --- | --- | --- | --- |

1. Saya dapat memperkenalkan diri secara singkat dalam Bahasa Inggris tanpa membawa catatan.

|  |  |  |  |
| --- | --- | --- | --- |

1. Saya merasa saya bisa berbicara dalam Bahasa Inggris tanpa harus terlalu bersusah payah.

|  |  |  |  |
| --- | --- | --- | --- |

1. Saya yakin bahwa saya akan dapat berusaha untuk membuat diri saya dipahami ketika berbicara dalam Bahasa Inggris.

|  |  |  |  |
| --- | --- | --- | --- |

1. Saya tahun saya bisa berusaha berbicara dalam Bahasa Inggris dengan menggunakan kata-kata yang saya tahu meskipun kata-kata tersebut tidak tepat.

|  |  |  |  |
| --- | --- | --- | --- |

1. Saya bisa berbicara tanpa merasa gugup.

|  |  |  |  |
| --- | --- | --- | --- |

1. Saya merasa baik-baik saja saat berbicara Bahasa Inggris di kelas yang jumlah siswanya seperti kelas ini.

|  |  |  |  |
| --- | --- | --- | --- |

1. Saya bisa berbicara Bahasa Inggris di depan umum. Saya bisa melakukannya.

|  |  |  |  |
| --- | --- | --- | --- |

1. Saya merasa lebih nyaman berbicara Bahasa Inggris di depan umum.

|  |  |  |  |
| --- | --- | --- | --- |

1. Saya bisa berbicara dalam Bahasa Inggris secara lebih lancar dari sebelumnya.

|  |  |  |  |
| --- | --- | --- | --- |

1. Saya merasa Bahasa Inggris saya lumayan baik

|  |  |  |  |
| --- | --- | --- | --- |

1. Saya tahu saya dapat berbicara Bahasa Inggris ketika berpidato jika saya sudah mempersiapkan diri.

|  |  |  |  |
| --- | --- | --- | --- |

1. Saya dapat berusaha berpidato jika saya sudah menyiapkan draftnya.

|  |  |  |  |
| --- | --- | --- | --- |

**Lampiran 2: Jawaban peserta untuk butir-butir kuesioner CA mereka**

1. Saya merasa tegang ketika presentasi.

|  |  |  |  |
| --- | --- | --- | --- |

1. Bahkan saat ini saya merasa trauma akibat kegagalan saya ketika melakukan presentasi.

|  |  |  |  |
| --- | --- | --- | --- |

1. Saya merasa khawatir bahwa saya mungkin menggunakan Bahasa Inggris yang aneh.

|  |  |  |  |
| --- | --- | --- | --- |

1. Karena saya sadar bahwa Bahasa Inggris saya buruk, sulit sekali bagi saya untuk berbicara dalam Bahasa Inggris dan saya kehilangan rasa percaya diri saya.

|  |  |  |  |
| --- | --- | --- | --- |

1. Saya merasa malu untuk berbicara di depan umum.

|  |  |  |  |
| --- | --- | --- | --- |

1. Saya merasa sedikit gugup jika Bahasa Inggris saya salah ketika berbicara di tempat umum.

|  |  |  |  |
| --- | --- | --- | --- |

1. Meskipun saya sudah beberapa kali berpidato, saya tetap merasa tegang.

|  |  |  |  |
| --- | --- | --- | --- |

1. Saya tidak pernah merasa cukup yakin terhadap diri saya sendiri ketika saya berbicara di kelas Bahasa Inggris saya.

|  |  |  |  |
| --- | --- | --- | --- |

1. Bagi saya, tidak memahami apa yang guru katakan dalam Bahasa Inggris adalah hal yang menakutkan.

|  |  |  |  |
| --- | --- | --- | --- |

1. Saya mulai merasa panik ketika saya harus berbicara tanpa persiapan di kelas Bahasa Inggris.

|  |  |  |  |
| --- | --- | --- | --- |

1. Saya merasa sangat sadar diri ketika berbicara Bahasa Inggris di depan siswa lain.

|  |  |  |  |
| --- | --- | --- | --- |

1. Saya merasa gugup dan bingung ketika saya berbicara dalam Bahasa Inggris di kelas Bahasa Inggris saya.

|  |  |  |  |
| --- | --- | --- | --- |

1. Saya merasa gugup ketika saya tidak memahami setiap kata yang dikatakan oleh guru bahasa.

|  |  |  |  |
| --- | --- | --- | --- |

1. Saya merasa kewalahan dengan jumlah aturan yang harus dipelajari ketika berbicara dalam Bahasa Inggris.

|  |  |  |  |
| --- | --- | --- | --- |

1. Saya gemetar ketika tahu bahwa saya akan ditunjuk guru di kelas Bahasa Inggris.

|  |  |  |  |
| --- | --- | --- | --- |

**LAMPIRAN A**

**Kuesioner**

Untuk butir-butir berikut, lingkari angka yang paling menggambarkan diri Anda (mulai dari 1 = Sangat Tidak Setuju sampai 5 = Sangat Setuju) .

1. Siapa nama Anda?
2. Kelas bahasa Inggris sekolah menengah saya mempertemukan kami dengan penutur asli Bahasa Inggris.

1 2 3 4 5

1. Saya bisa membaca dengan baik dalam Bahasa Inggris.

1 2 3 4 5

1. Saya memikirkan tata bahasa (grammar) sebelum saya berbicara.

1 2 3 4 5

1. Saya yakin bahwa saya tahu cara penggunaan *who, which, that, what, whatever, whoever, whichever*

1 2 3 4 5

1. Saya pandai mendengarkan Bahasa Inggris.

1 2 3 4 5

1. Saya yakin bahwa saya tahu bagaimana menyatakan perbandingan dalam Bahasa Inggris.

1 2 3 4 5

8a) Saya merasa percaya diri dengan kemampuan saya membaca dalam Bahasa Inggris.

1 2 3 4 5

8b) Mohon jelaskan jawaban Anda.

9a) Saya merasa percaya diri dengan kemampuan saya menulis dalam Bahasa Inggris.

1 2 3 4 5

9b) Mohon jelaskan jawaban Anda.

8a) Saya merasa percaya diri dengan kemampuan saya mendengarkan dalam Bahasa Inggris.

1 2 3 4 5

10b) Mohon jelaskan jawaban Anda.

9a) Saya merasa percaya diri dengan kemampuan saya berbicara dalam Bahasa Inggris.

1 2 3 4 5

11b) Mohon jelaskan jawaban Anda.

1. Saya yakin bahwa saya tahu cara menyusun kalimat dalam *present tense.*

1 2 3 4 5

1. Saya yakin bahwa saya tahu cara menggunakan conditional.

1 2 3 4 5

1. Saya bisa mendengar dengan baik dalam Bahasa Inggris.

1 2 3 4 5

1. Saya yakin bahwa saya tahu cara menggunakan *future perfect continuous tense. (I’ll have finished reading this book by the end of this month.)*

1 2 3 4 5

1. Saya bisa berbicara dengan baik dalam Bahasa Inggris.

1 2 3 4 5

1. Saya pandai menulis dalam Bahasa Inggris.

1 2 3 4 5

1. Saya yakin bahwa saya tahu cara membuat kalimat pasif dalam Bahasa Inggris.

1 2 3 4 5

1. Saya pandai berbicara dalam Bahasa Inggris

1 2 3 4 5

1. Saya yakin bahwa saya tahu cara menggunakan past perfect tense.

1 2 3 4 5

1. Saya merasa gugup ketika berbicara dalam bahasa Inggris dengan guru ELI?

1 2 3 4 5

1. Saya merasa gugup ketika berbicara dengan penutur asli selain guru ELI, misalnya dengan siswa pertukaran atau saat liburan?

1 2 3 4 5

1. Saya pandai membaca dalam Bahasa Inggris

1 2 3 4 5

1. Saya bisa menulis dengan baik dalam Bahasa Inggris.

1 2 3 4 5

1. Saya yakin bahwa saya tahu cara menyusun kalimat dalam *past tense*. (“ *He walked home*.”)

1 2 3 4 5

1. Saya yakin bahwa saya tahu bagaimana menggunakan *modals. (may, must, could, should, would, have to, be able to)*

1 2 3 4 5

**Lampiran B**

**Tingkatan penilaian verbal KEPT**

|  | **Pelafalan**  Pikirkan tentang:   - **Tingkat kata** - **Tingkat Kalimat: kemampuan untuk 'menyatukan' atau menghubungkan suara di dalam atau di antara kata-kata.** - **Tekanan, ritme, dan intonasi** - **Aksen** | **Kefasihan**  Pikirkan tentang:   - **Otomatisasi: kemampuan untuk merumuskan ucapan dengan cepat dan berbicara dengan lancar** - **Kecepatan bicara** - **Keraguan dan jeda** | **Leksis/Tata Bahasa**  Pikirkan tentang:   - **Bentuk tata bahasa yang benar** - **Kesesuaian kosakata** - **Menampilkan kemampuan untuk menggunakan (atau mencoba menggunakan) struktur tata bahasa dan kosa kata yang berbeda sesuai konteksnya.** - **Kolokasi dan pilihan kata yang benar** | **Keterampilan percakapan**  Pikirkan tentang:   - **Partisipasi dan kelancaran interaksi (bergantian, menanggapi orang lain, mengajukan pertanyaan dan memperkenalkan langkah baru, parafrase, *hedging*^[[2]](#footnote-2)^)** |
| --- | --- | --- | --- | --- |
|  | **Pelafalan yang tidak dapat diterima**   - Aksen yang sangat kental, yang akan menyebabkan gangguan dalam komunikasi - Hanya menggunakan fonologi dan ritme seperti katakana; kata-kata tidak tercampur bersama-sama | **Kefasihan yang tidak dapat diterima**   - Patah-patah ketika berbicara - Sering berhenti, sering tidak bisa dimengerti - Komunikasi hampir tidak mungkin dilakukan | **Penggunaan leksikal & tata bahasa yang tidak dapat diterima** | **Interaksi percakapan yang tidak dapat diterima**   - Tidak menunjukkan kesadaran terhadap adanya pembicara lain; dapat berbicara, tetapi tidak dengan cara seperti percakapan - Komunikasi tidak mungkin |
| **0**  **~ 0.5** |  |  | - Tidak ada bukti pengetahuan tata bahasa - Tahu beberapa kata, dan menggunakannya secara terpisah - Tidak dapat berbagi gagasan sederhana - Komunikasi tidak mungkin |  |
| **1.0**  **~ 1.5** | **Pelafalan yang buruk**   - Menggunakan pengucapan seperti Katakana; tidak memadukan kata-kata - Kemungkinan mengalami kesulitan memahami lawan bicara | **Kefasihan yang buruk**   - Tegang, lambat, *ucapan tidak wajar* - Sering meraba-raba kata-kata*tidak wajar* - Jeda panjang secara *tidak wajar* - Komunikasi sulit | **Penggunaan leksikal & tata bahasa yang buruk**   - Ada bukti keberadaan sejumlah pengetahuan tata bahasa yang sangat terbatas - Kosakata terbatas tetapi penggunaan tidak ahli - Sedikit atau tidak ada upaya pada kosakata atau tata bahasa yang kompleks | **Interaksi percakapan yang buruk**   - Tidak memulai interaksi - Sebagian besar menggunakan gaya monolog - Mungkin menunjukkan berbagi giliran bicara tingkat dasar tetapi tidak menghubungkan gagasan dengan baik, atau memberikan banyak penjelasan |
|  |  |  | - Gagasan dapat disampaikan, tetapi dengan kemungkinan kesulitan pemahaman |  |
| **2.0**  **~ 2.5** | **Pelafalan sedang**   - Belum menguasai beberapa suara bahasa Inggris yang sulit, tetapi sebagian besar dapat dimengerti oleh lawan bicara - Sering berupaya untuk memadukan kata-kata tetapi mungkin masih salah menekankan kata-kata | **Kefasihan cukup lancar**   - Bicaranya ragu-ragu; agak *tidak wajar* - Meraba-raba kata-kata secara *tidak wajar* dan kekosongan percakapan mungkin tetap ada, tetapi itu tidak sepenuhnya menghalangi komunikasi - Mungkin menggunakan ungkapan pengisi (filler) secara berlebihan, atau menunjukkan penggunaan *tidak wajar lainnya* | **Penggunaan leksikal & tata bahasa sedang**   - Terlalu bergantung pada sejumlah kecil tata bahasa dan kosa kata sederhana untuk mengekspresikan gagasan - Menunjukkan sedikit atau tidak ada bukti kemampuan untuk mengontrol tata bahasa atau kosa kata yang sulit | **Interaksi percakapan sedang**   - Kesadaran dalam bergiliran - Mempertahankan interaksi dengan menanggapi orang lain tanpa jeda yang tidak wajar atau berhenti secara tidak wajar - Menunjukkan persetujuan atau ketidaksetujuan yang berarti terhadap pendapat orang lain (setuju/tidak setuju, dll) |
| **3.0**  **~ 3.5** | **Penlafalan yang sangat baik**   - Mungkin tidak menguasai semua suara Bahasa Inggris, tetapi memiliki kontrol yang baik untuk penekanan dalam kalimat dan intonasi. - Aksen tidak mengganggu pemahaman; dapat memadukan kata-kata secara konsisten | **Kefasihan yang sangat baik**   - Penyalahgunaan pengisi percakapan (filler) sesekali, meraba-raba dan memperbaiki ungkapan mungkin masih terlihat, tetapi tidak terlalu mengganggu pendengar. | **Penggunaan leksikal & tata bahasa yang sangat baik**   - Menunjukkan bukti kemampuan untuk mengontrol tata bahasa atau kosa kata yang sulit dan upaya untuk menggunakan berbagai bentuk. - Mungkin terus membuat kesalahan, tetapi tetap dapat dipahami. | **Interaksi percakapan yang sangat baik**   - Tampil percaya diri - Merespon dengan tepat kepada orang lain - Dapat mengarahkan percakapan - Menunjukkan kemampuan untuk menegosiasikan makna dengan cepat dan alami - Mungkin mulai menggunakan parafrase atau klarifikasi sebagai sarana untuk perancah bagi lawan bicara dengan kemampuan di tingkat yang lebih rendah |
| **4**  **~**  **?** | **Pelafalan yang sangat baik**   - Tampaknya telah menguasai banyak tata suara bahasa Inggris - Aksen tidak menghalangi komunikasi | **Kefasihan yang sangat baik**   - Percakapan berjalan lancar, dengan sedikit hambatan. - Menggunakan pengisi percakapan (filler), penanda, potongan leksikal secara efektif. - Meraba-raba kata dapat terjadi, tetapi tampak alami & lancar. | **Penggunaan tata bahasa & kosa kata yang sangat baik**   - Menunjukkan kontrol yang sangat baik terhadap berbagai tata bahasa dan kosa kata - Kesalahan mungkin masih terjadi, tetapi tidak akan menghalangi makna - Item leksikal yang dipotong, seperti idiom dan kolokasi mungkin ada dan digunakan dengan benar | **Interaksi percakapan yang sangat baik**   - Sangat percaya diri dan alami - Mungkin meminta orang lain untuk memperluas pandangan - Bernegosiai, menahan dan melepaskan giliran berbicara dengan tepat - Menjelaskan bagaimana gagasan sendiri dan gagasan orang lain saling terkait, berinteraksi dengan lancar |

**THE FORMAT GIVEN TO PARTICIPANTS:**

EFN Questionairre Pre

Kami tim peneliti dari Fakultas Keperawatan Unpad sedang melakukan penelitian untuk menguji Self-Perceived Communication Competence (SPCC) dan Communication Apprehension (CA) mahasiswa dalam mempelajari bahasa inggris dalam keperawatan.

Kuesioner tersebut mengkaji persepsi diri mahasiswa terkait kompetensi dalam berkomunikasi dengan menggunakan bahasa inggris.

Kami mengundang Anda untuk berpartisipasi dalam penelitian ini, karena anda merupakan mahasiswa Fakultas Keperawatan Unpad.

Anda hanya akan mengisi kuesioner, sehingga kecil kemungkinan akan menimbulkan risiko yang merugikan.

Manfaat langsung yang bisa anda dapatkan dengan menjadi responden penelitian ini adalah dapat membantu anda untuk mengukur berdasarkan persepsi diri kompetensi anda dalam berbahasa inggris, sehingga hasi penelitian ini dapat enjadi dasar bagi tim pengajar untuk mengembangakan metode pembelajaran yang tepat bagi anda untuk dapat meraih capaian pembelajaran yang diharapkan.

Keikutsertaan Anda dalam penelitian ini bersifat sukarela, jadi Anda dapat memutuskan untuk berpartisipasi atau sebaliknya.

Identitas anda akan dijaga kerahasiaannya, informasi yang diberikan hanya akan digunakan untuk penelitian.

Apabila ada hal-hal yang ingin ditanyakan anda dapat menghubungi: Hartiah Haroen, SKp., MNg., M.Kes., AIFO, Phd(c)

No. Hp: +62 812-9454-3843 email: [hartiah@unpad.ac.id](mailto:hartiah@unpad.ac.id)

* Required

1. **Email** *
2. **Nama** *
3. **NPM** *
4. **Jenis Kelamin** *

*Mark only one oval.*

Laki-laki Perempuan

1. **Usia (dalam tahun)** *

PERNYATAAN PERSETUJUAN

Saya telah membaca atau memperoleh penjelasan, sepenuhnya menyadari, mengerti, dan memahami tentang tujuan, manfaat, dan risiko yang mungkin timbul dalam penelitian.

1. **Oleh karena itu, saya dengan sukarela memilih untuk ikut serta dalam** * **penelitian ini tanpa tekanan/paksaan siapapun, maka saya setuju/tidak setuju*) ikut dalam penelitian ini**

*Mark only one oval.*

Setuju Tidak setuju

Kuesioner Penelitian

1. **Apakah anda pernah atau sedang mengikuti kursus bahasa inggris** *

*Mark only one oval.*

Pernah

Sedang menjalani

Belum pernah

1. **Apakah anda senang belajar bahasa inggris?** *

*Mark only one oval.*

Tidak Ya

1. **Apakah anda sering membaca teks berbahasa inggris** *

*Mark only one oval.*

Tidak pernah Kadang-kadang Sering

1. **Berapakah nilai IPK anda saat ini** *

Section: Self-Perceived Communication Competence (SPCC) - 1

1. **Saya dapat memberikan petunjuk arah ke suatu tempat dalam Bahasa** *

Inggris kepada teman yang duduk di sebelah saya.

*Mark only one oval.*

1 2 3 4

Sangat tidak setuju Sangat Setuju

1. **Saya dapat menerjemahkan ungkapan verbal dalam Bahasa Indonesia** *

ke Bahasa Inggris di kelompok saya.

*Mark only one oval.*

1 2 3 4

Sangat tidak setuju Sangat setuju

1. **Saya dapat bermain peran dalam Bahasa Inggris di depan kelas** *

(misalnya berperan menjadi perawat yang sedang melakukan anamnesa)

*Mark only one oval.*

1 2 3 4

Sangat tidak setuju Sangat setuju

1. **Saya dapat memperkenalkan diri secara singkat dalam Bahasa Inggris** *

tanpa membawa catatan

*Mark only one oval.*

1 2 3 4

Sangat tidak setuju Sangat setuju

terlalu bersusah payah

*Mark only one oval.*

1 2 3 4

Sangat tidak setuju Sangat setuju

1. **Saya yakin bahwa saya akan dapat berusaha untuk membuat diri saya** *

dipahami ketika berbicara dalam Bahasa Inggris

*Mark only one oval.*

1 2 3 4

Sangat tidak setuju Sangat setuju

1. **Saya tahu saya bisa berusaha berbicara dalam Bahasa Inggris dengan** * **menggunakan kata-kata yang saya tahu meskipun kata-kata tersebut tidak tepat**

*Mark only one oval.*

1 2 3 4

Sangat tidak setuju Sangat setuju

1. **Saya bisa berbicara tanpa merasa gugup** *

*Mark only one oval.*

1 2 3 4

Sangat tidak setuju Sangat setuju

yang jumlah siswanya seperti kelas ini

*Mark only one oval.*

1 2 3 4

Sangat tidak setuju Sangat setuju

1. **Saya bisa berbicara Bahasa Inggris di depan umum. Saya bisa** *

melakukannya.

*Mark only one oval.*

1 2 3 4

Sangat tidak setuju Sangat setuju

1. **Saya merasa lebih nyaman berbicara Bahasa Inggris di depan umum** *

*Mark only one oval.*

1 2 3 4

Sangat tidak setuju Sangat setuju

1. **Saya bisa berbicara dalam Bahasa Inggris secara lebih lancar dari** *

sebelumnya

*Mark only one oval.*

1 2 3 4

Sangat tidak setuju Sangat setuju

1. **Saya tahu saya dapat berbicara Bahasa Inggris ketika berpidato jika** *

saya sudah mempersiapkan diri

*Mark only one oval.*

1 2 3 4

Sangat tidak setuju Sangat setuju

1. **Saya dapat berusaha menjelaskan sesuatu jika saya sudah** *

menyiapkan draftnya

*Mark only one oval.*

1 2 3 4

Sangat tidak setuju Sangat setuju

Section: Self-Perceived Communication Competence (SPCC) - 2

1. **Saya bisa membaca dengan baik dalam Bahasa Inggris** *

*Mark only one oval.*

1 2 3 4

Sangat tidak setuju Sangat setuju

1. **Saya yakin bahwa saya tahu cara penggunaan who, which, that, what,** *

whatever, whoever, whichever

*Mark only one oval.*

1 2 3 4

Sangat tidak setuju Sangat setuju

1. **Saya pandai mendengarkan Bahasa Inggris** *

*Mark only one oval.*

1 2 3 4

Sangat tidak setuju Sangat setuju

1. **Saya yakin bahwa saya tahu bagaimana menyatakan perbandingan** *

dalam Bahasa Inggris

*Mark only one oval.*

1 2 3 4

Sangat tidak setuju Sangat setuju

Bahasa Inggris

*Mark only one oval.*

1 2 3 4

Sangat tidak setuju Sangat setuju

1. **Saya merasa percaya diri dengan kemampuan saya menulis dalam** *

Bahasa Inggris

*Mark only one oval.*

1 2 3 4

Sangat tidak setuju Sangat setuju

1. **Saya merasa percaya diri dengan kemampuan saya mendengarkan** *

dalam Bahasa Inggris

*Mark only one oval.*

1 2 3 4

Sangat tidak setuju Sangat setuju

1. **Saya merasa percaya diri dengan kemampuan saya berbicara dalam** *

Bahasa Inggris

*Mark only one oval.*

1 2 3 4

Sangat tidak setuju Sangat setuju

1. Saya yakin bahwa saya tahu cara menyusun kalimat dalam present tense

*Mark only one oval.*

1 2 3 4

Sangat tidak setuju Sangat setuju

1. **Saya yakin bahwa saya tahu cara menggunakan conditional** *

*Mark only one oval.*

1 2 3 4

Sangat tidak setuju Sangat setuju

1. **Saya bisa mendengar dengan baik dalam Bahasa Inggris** *

*Mark only one oval.*

1 2 3 4

Sangat tidak setuju Sangat setuju

1. **Saya yakin bahwa saya tahu cara menggunakan future perfect** *

continuous tense. (I’ll have finished reading this book by the end of this month.)

*Mark only one oval.*

1 2 3 4

Sangat tidak setuju Sangat setuju

1. **Saya pandai menulis dalam Bahasa Inggris** *

*Mark only one oval.*

1 2 3 4

Sangat tidak setuju Sangat setuju

1. **Saya yakin bahwa saya tahu cara membuat kalimat pasif dalam Bahasa** *

Inggris

*Mark only one oval.*

1 2 3 4

Sangat tidak setuju Sangat setuju

1. **Saya pandai berbicara dalam Bahasa Inggris** *

*Mark only one oval.*

1 2 3 4

Sangat tidak setuju Sangat setuju

1. **Saya merasa gugup ketika berbicara dengan penutur asli (native** *

speaker) misalnya dengan dosen tamu dari luar negeri

*Mark only one oval.*

1 2 3 4

Sangat tidak setuju Sangat setuju

1. **Saya pandai membaca dalam Bahasa Inggris** *

*Mark only one oval.*

1 2 3 4

Sangat tidak setuju Sangat setuju

1. **Saya bisa menulis dengan baik dalam Bahasa Inggris** *

*Mark only one oval.*

1 2 3 4

Sangat tidak setuju Sangat setuju

(“ He walked home.”)

*Mark only one oval.*

1 2 3 4

Sangat tidak setuju Sangat setuju

1. **Saya yakin bahwa saya tahu bagaimana menggunakan modals. (may,** *

must, could, should, would, have to, be able to)

*Mark only one oval.*

1 2 3 4

Sangat tidak setuju Sangat setuju

Section: Communication Apprehension (CA)

1. **Saya merasa tegang ketika presentasi** *

*Mark only one oval.*

1 2 3 4

Sangat setuju Sangat tidak setuju

1. **Bahkan saat ini saya merasa trauma akibat kegagalan saya ketika melakukan presentasi**

*Mark only one oval.*

1 2 3 4

Sangat setuju Sangat tidak setuju

Inggris yang aneh

*Mark only one oval.*

1 2 3 4

Sangat setuju Sangat tidak setuju

1. **Karena saya sadar bahwa Bahasa Inggris saya buruk, sulit sekali bagi** * **saya untuk berbicara dalam Bahasa Inggris dan saya kehilangan rasa percaya diri saya**

*Mark only one oval.*

1 2 3 4

Sangat setuju Sangat tidak setuju

1. **Saya merasa malu untuk berbicara di depan umum** *

*Mark only one oval.*

1 2 3 4

Sangat setuju Sangat tidak setuju

1. **Saya merasa sedikit gugup jika Bahasa Inggris saya salah ketika** *

berbicara di tempat umum

*Mark only one oval.*

1 2 3 4

Sangat setuju Sangat tidak setuju

depan kelas, saya tetap merasa tegang

*Mark only one oval.*

1 2 3 4

Sangat setuju Sangat tidak setuju

1. **Saya tidak pernah merasa cukup yakin terhadap diri saya sendiri ketika** *

saya berbicara di kelas Bahasa Inggris saya

*Mark only one oval.*

1 2 3 4

Sangat setuju Sangat tidak setuju

1. **Bagi saya, tidak memahami apa yang dosen katakan dalam Bahasa** *

Inggris adalah hal yang menakutkan

*Mark only one oval.*

1 2 3 4

Sangat setuju Sangat tidak setuju

1. **Saya mulai merasa panik ketika saya harus berbicara tanpa persiapan** *

di kelas Bahasa Inggris

*Mark only one oval.*

1 2 3 4

Sangat setuju Sangat tidak setuju

depan mahasiswa lain

*Mark only one oval.*

1 2 3 4

Sangat setuju Sangat tidak setuju

1. **Saya merasa gugup dan bingung ketika saya berbicara dalam Bahasa** *

Inggris di kelas Bahasa Inggris saya

*Mark only one oval.*

1 2 3 4

Sangat setuju Sangat tidak setuju

1. **Saya merasa gugup ketika saya tidak memahami setiap kata yang** *

dikatakan oleh Dosen bahasa inggris

*Mark only one oval.*

1 2 3 4

Sangat setuju Sangat tidak setuju

1. **Saya merasa kewalahan dengan jumlah aturan yang harus dipelajari** *

ketika berbicara dalam Bahasa Inggris

*Mark only one oval.*

1 2 3 4

Sangat setuju Sangat tidak setuju

Bahasa Inggris

*Mark only one oval.*

1 2 3 4

Sangat setuju

Sangat tidak setuju

This content is neither created nor endorsed by Google.

[Forms](https://www.google.com/forms/about/?utm_source=product&utm_medium=forms_logo&utm_campaign=forms)

1. Hedging is the softening of facts using probability such as in *Patients may experience hallucination* instead of *Patients experience hallucination (trans.)* [↑](#footnote-ref-1)
2. Hedging adalah memperhalus pernyataan fakta dengan menggunakan kemungkinan seperti dalam kalimat *Patients may experience hallucination* sebagai ganti dari *Patients experience hallucination (trans.)* [↑](#footnote-ref-2)
